# Cervical Artery Dissection and Choosing Appropriate Therapy

**DOI:** 10.5811/cpcem.2017.3.33296

**Published:** 2017-07-06

**Authors:** Jonathan T. Lau, John S. Hunt, David I. Bruner, Andrea L. Austin

**Affiliations:** * Naval Medical Center, Department of Emergency Medicine, San Diego, California; † Scripps Mercy, Department of Emergency Medicine, San Diego, California

## Abstract

Cervical artery dissection is a common cause of stroke in young adults. This may result from head and neck trauma; it can also occur spontaneously or secondary to genetic connective tissue or vascular disorders. Neurologic symptoms arise as a result of thromboembolism and hypoperfusion causing cerebral ischemia. We present a case of a previously healthy male who was found to have a cervical internal carotid artery dissection and the decision to use antiplatelet therapy instead of anticoagulation to prevent stroke. Data is lacking regarding the efficacy of one therapy over the other.

## INTRODUCTION

Cervical artery dissection is a common cause of stroke in young adults with an incidence of 2.6–2.9 per 100,000 patients. It is classified based on the artery involved (vertebral vs. carotid) and location (intracranial vs. extracranial). The most common dissection is an extracranial internal carotid artery (ICA) dissection. Dissection is defined by the separation of arterial wall layers, which creates a false lumen allowing blood to escape and expand. This may result from head and neck trauma or can occur spontaneously or secondary to genetic connective tissue or vascular disorders, such as Ehlers-Danlos syndrome, Marfan syndrome and most commonly fibromuscular dysplasia. Neurologic symptoms arise as a result of thromboembolism and hypoperfusion causing cerebral ischemia. Mass effect of the dissecting artery may also cause local nerve root compression resulting in Horner’s syndrome.

## CASE REPORT

We present a case of a 30-year-old previously healthy male who presented to the emergency department (ED) with complaints of a new gradual onset left-sided throbbing headache with transient right-sided paresthesias and a right hemianopsia that occurred at rest. There was no associated weakness or other focal neurologic deficits. His symptoms spontaneously resolved on arrival to the ED. He reported recent chiropractic manipulation of the cervical spine one week prior to presentation. He also noted remote trauma, having been involved in a motorcycle accident approximately nine months prior sustaining a left clavicle fracture that was initially treated non-operatively. However, he required surgical repair due to malunion. At the time of ED presentation, his neurologic exam was unremarkable.

**Image 1:**
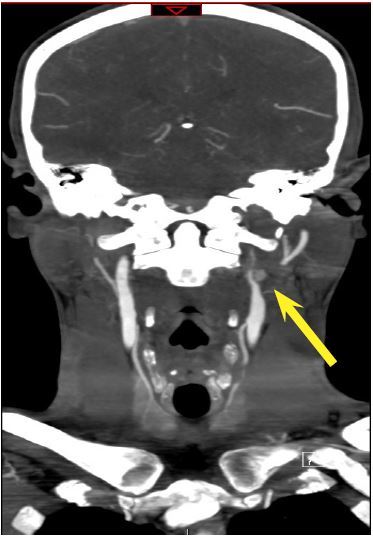
Computerized tomography angiography (CTA) coronal view demonstrating the dissection with the pseudoaneurysm (yellow arrow).

**Image 2:**
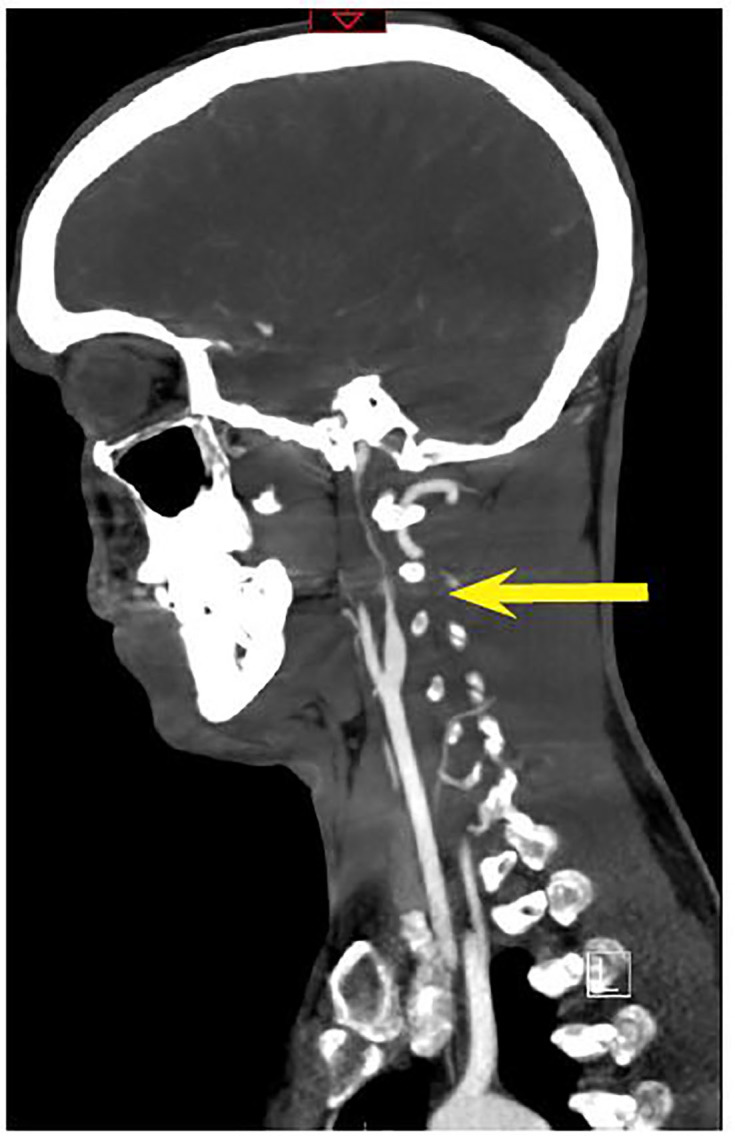
CTA sagittal view showing “carotid string sign” (yellow arrow) referring to the thin string of intravenous contrast material distal to the stenotic focus in the internal carotid artery.

Non-contrast head computerized tomography (CT) was unremarkable. CT angiography (CTA) of the head and neck was performed because of his reported transient neurologic deficits, and it revealed a left cervical ICA dissection 1.7cm from the carotid bifurcation extending to the base of the skull and a 7mm pseudoaneurysm near the proximal aspect of the dissection (Image 1 and 2). Both neurology and neurosurgery were consulted, and there was no consensus on stroke prophylaxis.

**Image 3:**
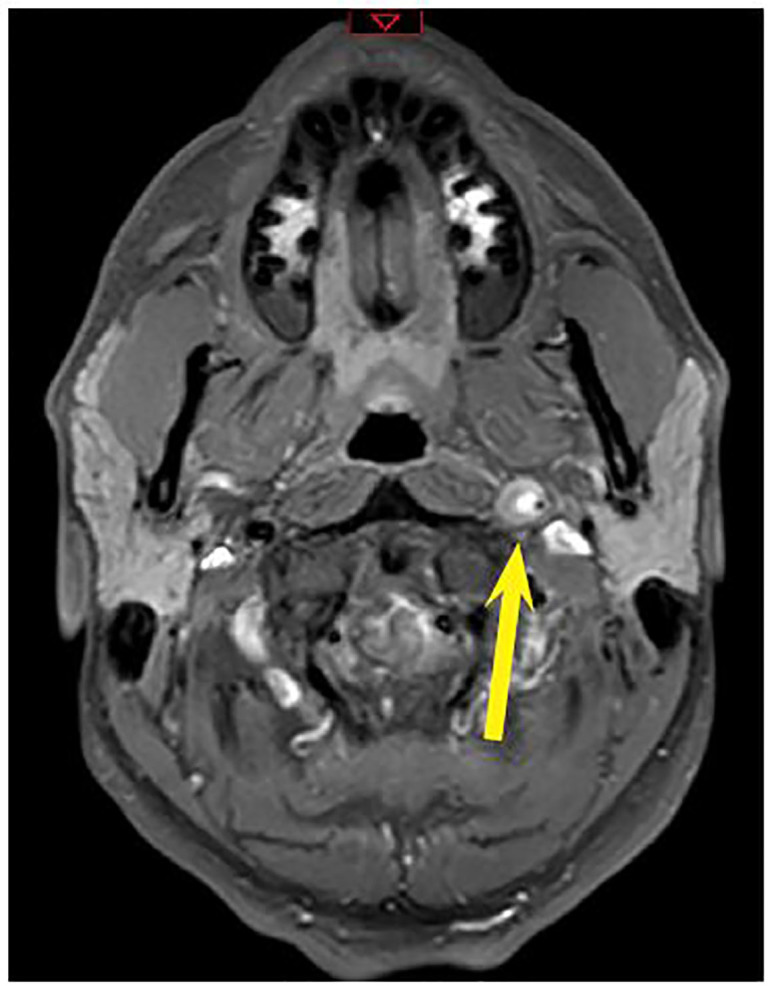
Magnetic resonance imaging (MRI) angiography T1 fat-saturated transverse view showing left internal carotid artery with significantly diminished lumen size and showing enhancement of thrombus (yellow arrow).

**Image 4:**
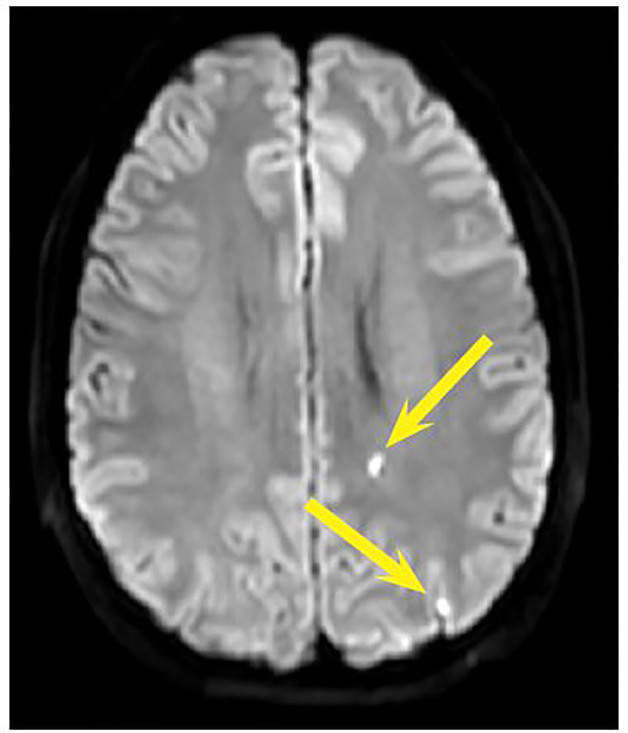
MRI transverse view showing areas with small ischemic lesions (yellow arrows).

The patient was ultimately started on oral aspirin 325mg daily and atorvastatin 80mg daily and admitted to the medical intensive care unit. Magnetic resonance imaging (MRI) with angiography of the head and neck were subsequently performed revealing no large perfusion defects (Image 3 and 4). Angiogram was performed, further demonstrating the left ICA dissection and pseudoaneurysm. Two ICA stents were placed given concerns for the patient’s long-term risk for stroke. He was then transitioned to dual antiplatelet therapy with clopidogrel and aspirin and discharged home. The patient had follow-up weeks later and remained asymptomatic.

## DISCUSSION

Cervical artery dissection usually presents with a combination of transient ischemic attack or ischemic stroke, headache, and neck pain. Reportedly, 56% of patients present with symptoms of cerebral ischemia and 25% with Horner’s syndrome. Head and neck pain are the most common symptoms of cervical artery dissection, found in 60%–90% of cases, but only 20% have sudden thunderclap headaches. Other presentations include tinnitus and scalp tenderness. The mean age of occurrence is approximately 45 years with a slightly higher male predominance (53–57%). Risk of recurrent stroke is 2–3%, typically occurring in the first two weeks after dissection.

Associations between cervical spine manipulation (CSM) and cervical artery dissection have been described in case reports and case control studies. In 2016, a systematic review and meta-analysis was published that concluded the quality of data on this relationship is low and there is no convincing evidence to support a causal link between CSM and cervical artery dissection. It concluded that associations were likely biased by the strong possibility that patients with early dissection-related symptoms, such as neck pain, seek chiropractic care prior to developing a stroke. With these studies in mind the American Heart Association and American Stroke Association (AHA/ASA) released a scientific statement, updated in November 2016, that while incidence of cervical artery dissection in CSM is probably low and causality remains difficult to prove, physicians should consider the possibility of cervical artery dissection in this patient population and inform patients of the possible connection.

Population Health Research Capsule

What do we already know about this clinical entity?

Cervical artery dissection is a common cause of stroke in young adults and can occur spontaneously, after trauma, or due to a connective tissue or vascular disorder.

What makes this presentation of disease reportable?

This patient presented with symptoms of a transient ischemic attack and had a history of recent trauma to the left neck and chiropractic manipulation.

What is the major learning point?

Either antiplatelet or anticoagulation medications should be initiated promptly – evidence is limited supporting efficacy of one over the other.

How might this improve emergency medicine practice?

Recognition of cervical artery dissection and initiating appropriate therapy early may reduce the potential for any permanent or prolonged neurologic dysfunction.

On presentation, standard approaches to management should be performed including blood pressure regulation, fluid administration, glycemic control, and other correction of metabolic derangements, with the primary goal in treatment of cervical artery dissection to prevent stroke. Treatment is sometimes started in the ED with antithrombotic or anticoagulation medication. However, research is limited regarding the efficacy of one therapy over the other. In 2010, a Cochrane review showed no randomized control trials comparing either antiplatelet or anticoagulant drugs with control or directly comparing them to each other, concluding that there was no evidence to support their routine use or any significant difference in efficacy in extracranial cervical artery dissection. In 2015, a meta-analysis of 38 (non-randomized) studies showed no significant difference between antiplatelet versus anticoagulation therapy with respect to death or disability. The Cervical Artery Dissection in Stroke Study (CADISS Trial) randomized 250 patients within seven days of stroke onset to antiplatelet or anticoagulation therapy, which showed no difference in stroke risk. A major limitation of this study was that it was underpowered given the low stroke recurrence rates in both groups. It is estimated that a trial with adequate power to show any potential difference would require approximately 4,800 patients in each treatment group. Conducting a study requiring such a large cohort with its low recurrence rates is impractical.

The AHA 2011 guidelines state that the relative efficacy of anticoagulation versus antiplatelet therapy is unknown, and antithrombotic treatment was recommended for three to six months for those who sustain a stroke or transient ischemic attack. The same year, an executive summary released from the American College of Cardiology Foundation and the ASA made Class IIa recommendations to initiate treatment with anticoagulation followed by antiplatelet therapy.

## CONCLUSION

This case highlights the need to have a high index of suspicion regarding cervical artery dissection after remote trauma. There is limited evidence to suggest a causal link between cervical spinal manipulation and cervical artery dissection. Symptoms may be mistaken for a migraine headache, musculoskeletal neck pain, or cerebral infarction/hemorrhage. MRI angiography, CTA, or angiography confirm the diagnosis and should be considered as part of the diagnostic workup in the ED when there are even transient or subjective neurologic complaints. Until there is further evidence to support one antithrombotic therapy over the other, decisions regarding anticoagulation versus antiplatelet therapy can be made in conjunction with the consulting physician and performed on a case-by-case basis.
